# The role of three-dimensional printed models of skull in anatomy education: a randomized controlled trail

**DOI:** 10.1038/s41598-017-00647-1

**Published:** 2017-04-03

**Authors:** Shi Chen, Zhouxian Pan, Yanyan Wu, Zhaoqi Gu, Man Li, Ze Liang, Huijuan Zhu, Yong Yao, Wuyang Shui, Zhen Shen, Jun Zhao, Hui Pan

**Affiliations:** 1 Department of Endocrinology, Endocrine Key Laboratory of Ministry of Health, Peking Union Medical College Hospital (PUMCH), Chinese Academy of Medical Sciences & Peking Union Medical College (CAMS & PUMC), Beijing, 100730 China; 2 National Virtual Simulation Laboratory Education Center of Medical Sciences, PUMCH, CAMS & PUMC, Beijing, 100730 China; 3 Eight-year Program of Clinical Medicine, PUMCH, CAMS & PUMC, Beijing, 100730 China; 4 Dept. of Neurosurgery, PUMCH, CAMS & PUMC, Beijing, 100730 China; 5 College of Information Science and Technology, Beijing Normal University, Beijing, 100875 China; 6 The State Key Laboratory of Management and Control for Complex Systems, Institute of Automation, Chinese Academy of Sciences (CASIA), Beijing, 100190 China; 7 Cloud Computing Center, Chinese Academy of Sciences, Dongguan, 523808 China; 8 Dept. of Education, PUMCH, CAMS & PUMC, Beijing, 100730 China

## Abstract

Three-dimensional (3D) printed models represent educational tools of high quality compared with traditional teaching aids. Colored skull models were produced by 3D printing technology. A randomized controlled trial (RCT) was conducted to compare the learning efficiency of 3D printed skulls with that of cadaveric skulls and atlas. Seventy-nine medical students, who never studied anatomy, were randomized into three groups by drawing lots, using 3D printed skulls, cadaveric skulls, and atlas, respectively, to study the anatomical structures in skull through an introductory lecture and small group discussions. All students completed identical tests, which composed of a theory test and a lab test, before and after a lecture. Pre-test scores showed no differences between the three groups. In post-test, the 3D group was better than the other two groups in total score (cadaver: 29.5 [IQR: 25–33], 3D: 31.5 [IQR: 29–36], atlas: 27.75 [IQR: 24.125–32]; p = 0.044) and scores of lab test (cadaver: 14 [IQR: 10.5–18], 3D: 16.5 [IQR: 14.375–21.625], atlas: 14.5 [IQR: 10–18.125]; p = 0.049). Scores involving theory test, however, showed no difference between the three groups. In this RCT, an inexpensive, precise and rapidly-produced skull model had advantages in assisting anatomy study, especially in structure recognition, compared with traditional education materials.

## Introduction

Anatomy is the basis of modern medicine. It is one of the most complicated courses in medical curriculum due to the vast levels of knowledge needed and demands for spatial imagination. Cadaveric dissection is an indispensable part of anatomy, and is superior to two-dimensional (2D) atlases in facilitating knowledge acquisition. However, cadaveric dissection has always been associated with ethical concerns^1, 2^, difficulties and potential risks of preservation and disposal of specimens^3^. Further, shortage of donors is another limitation associated with cadaveric dissection in some countries^2^.

Three-dimensional (3D) printing was first described by Charles W. Hull in 1986, and has been extensively used worldwide over the past 30 years^4^. Due to its precise reconstruction of intricate anatomical structures, there is an increasing use of 3D printing in medicine, ranging from basic anatomy to surgical practice and advanced research application. Educational models including bone^5^, skull^6^, lateral ventricles^7^, kidney^8^, liver^9^, duodenum^10^, heart^11^, and cerebral aneurysm^12^, have been constructed using 3D printing technology. High-quality models with efficiency equal to or better than cadavers, are promising tools in resolving challenges associated with ethics and hygiene associated with dissection. However, assessment of 3D models varies significantly, and is mostly limited to subjective evaluation. Search in PubMed for “Randomized controlled trial” and “three-dimensional printing” showed 12 items, out of which only three randomized controlled trials (RCT) compared the learning efficiency of 3D-printed models with cadavers or atlas^13–15^. Additional RCTs are needed to confirm the role of 3D printing in medical education.

Structure of skull is always one of the most complicated areas of anatomy. Skull base models were used for endoscopic training^6^, and education in temporal bone anatomy^16^. However, no precise 3D printed skull models that focus on basicranial structures are available.

We generated 3D skull models, with each piece of skull bone colored differently, using data collected from computed tomography (CT). To evaluate the learning efficiency with 3D printed models of skull, we conducted an RCT comparing 2D atlases, cadaveric skulls and 3D printed skulls.

## Materials and Methods

### Skull models based on three-dimensional printing technology

**Figure 1:**
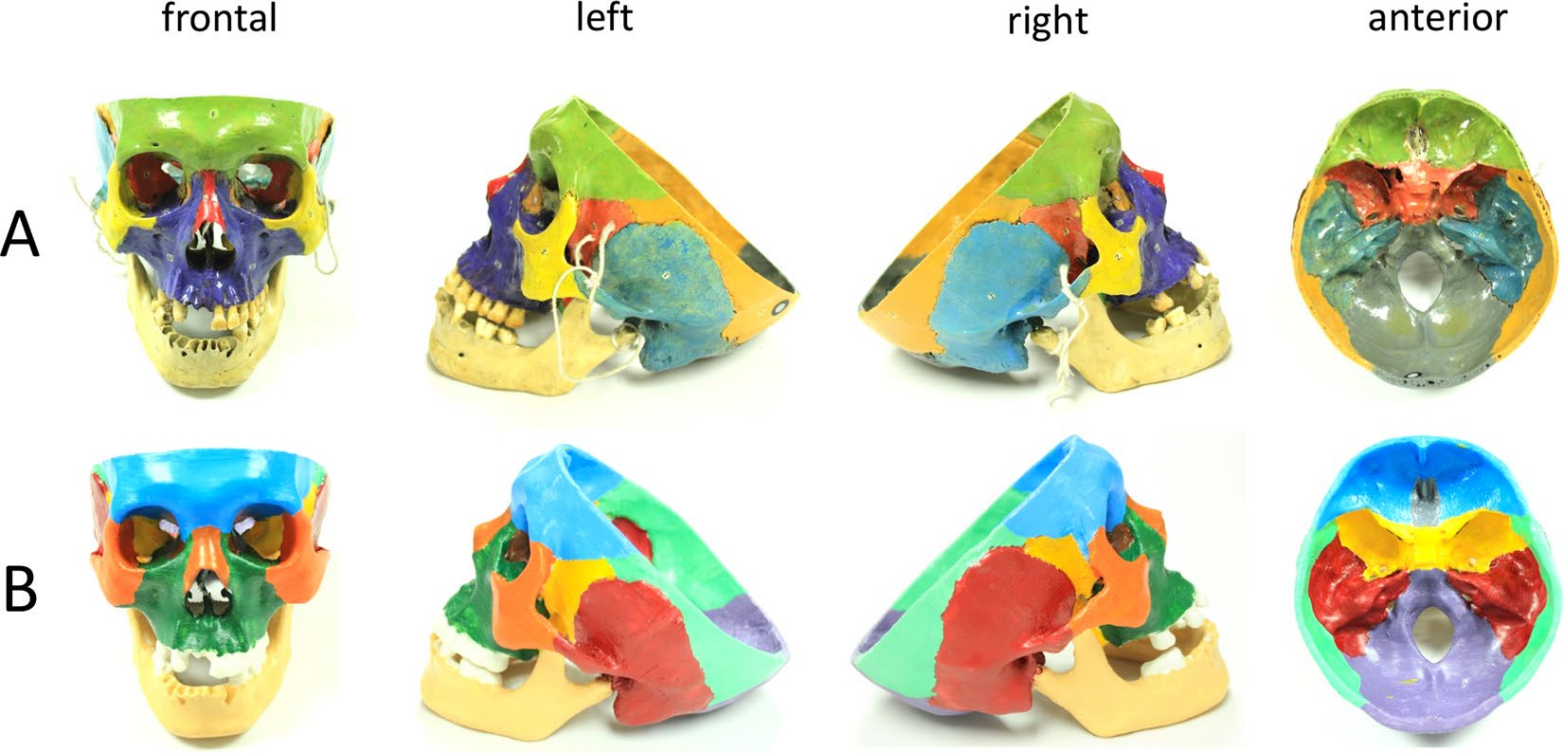
Photos of cadaveric skull and 3D-printed skull. (**A**) Cadaveric skull is showed in frontal, left, right and anterior views, respectively. Another four cadaveric skulls along with this one are used as teaching material for group 1. (**B**) 3D-printed skull is showed in frontal, left, right and anterior views, respectively. Five same 3D-printed skulls are used as teaching material for group 2.

**Figure 2:**
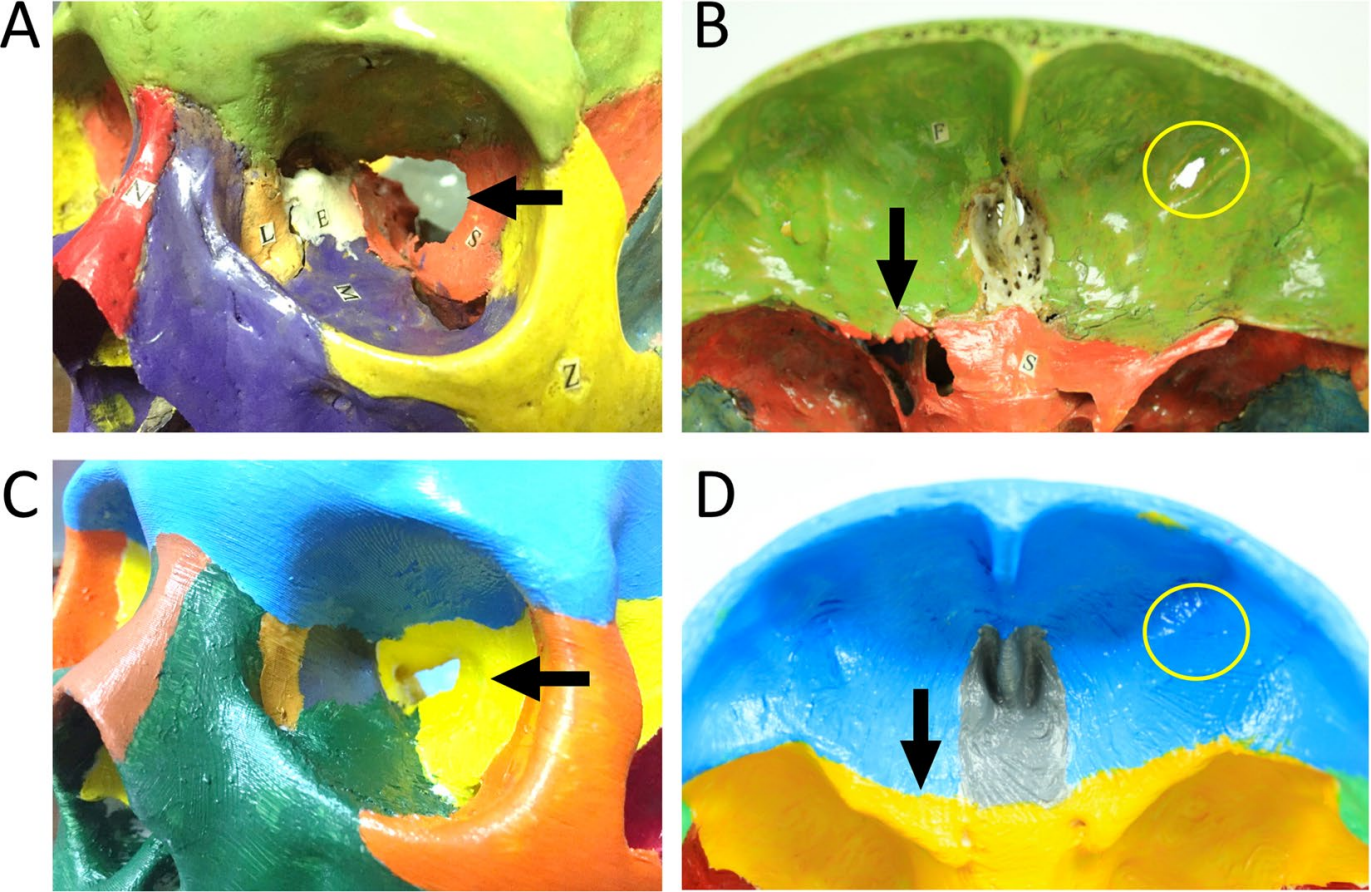
Comparison between the repaired structures of cadaveric skull and printed skull. (**A**,**C**) Orbit of cadaveric and 3D-printed skull, respectively. (**B**,**D**) part of anterior view of cadaveric and 3D-printed skull, respectively. Comparison of superior orbital fissure (black arrows in (**A**,**C**)), anterior clinoid process (black arrows in (**B**,**D**)), and small holes on frontal bone (yellow circle in (**B**,**D**)) between cadaveric skulls and 3D-printed skulls are shown.

We selected a most intact cadaveric skull in color (Fig. 1A), including clear anatomical basicranial structures from the Department of Anatomy in Peking Union Medical College (PUMC). CT scan of the cadaveric skull was obtained in the axial plane, with a slice width of 1 mm, a helical pitch of 0.9, and an image production interval of 1 mm. Scan data were exported to a DICOM file, and converted to STL (STereoLithography) file by commercial Materialise’s interactive medical image control system (Mimics 17.0). Several missing or damaged structures were found in the cadaveric skull as well as the initial 3D model, including anterior clinoid process, superior orbital fissure, and holes in the frontal bone (Fig. 2A). Therefore, we repaired the corresponding areas in the STL file, using the Geomagic Studio software and 3ds Max software (2014), and reviewed the STL file using Meshmixer before printing. A 3D printer designated as Ultimaker 2 (China, 2016, $500) based on fused deposition modeling technology was used for printing. Materials include white polylactic acid (PLA) with a diameter of 1.75 mm, and a layer thickness of 0.1 mm. Each piece of skull bone was painted using different colors (Fig. 1A) to assist in structure learning and minimize the differences between the three types of intervention. The repaired structures of cadaveric and printed skulls are compared (Fig. 2). The printing and painting of each skull lasted 28 h and 3 h, respectively. Raw materials for each skull cost approximately $14.

### Participants

Participants included third-year medical students at PUMC, who completed their pre-medical study and not yet introduced to anatomy curriculum. The study was announced by a grade counsellor three days before the trial, and 79 out of 80 students in this grade entered the trial voluntarily. All the participants completed the trial, without loss to follow-up.

### Ethical approval

No personal information was collected. Ethical approval was obtained from the Institutional Review Board of the Institute of Basic Medical Sciences, Chinese Academy of Medical Sciences (Project No: 009-2014). All participants completed written informed consent. Study methods were performed in accordance with approved guidelines.

### Study design

**Figure 3:**
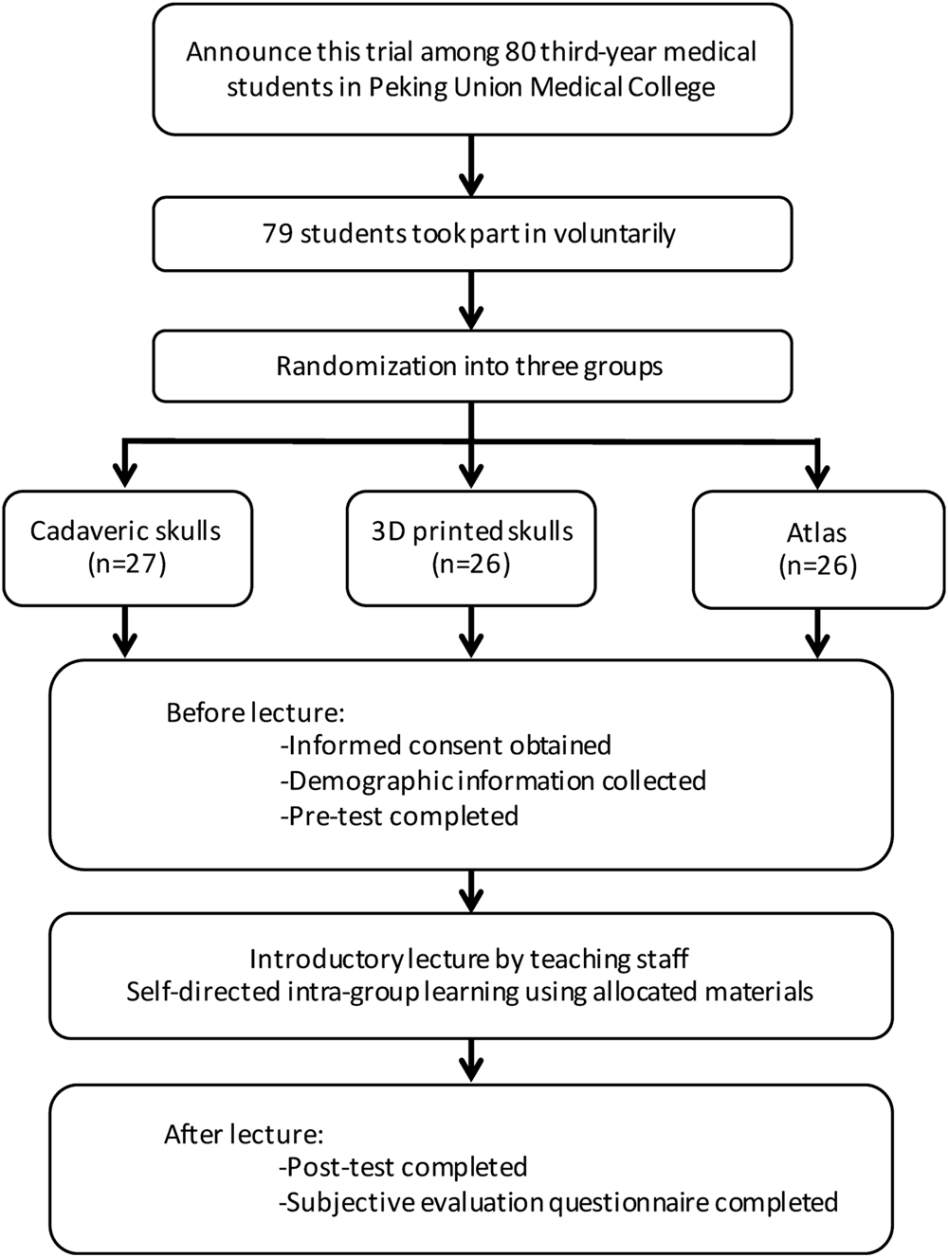
Flowchart of study design.

An RCT was designed to compare the learning efficiency of basicranial structures using three different learning materials separately. The flowchart of the study is displayed in Fig. 3. The 79 participants were randomly assigned to three groups by drawing lots. Each participant randomly selected a note marked with 1, 2 or 3. Twenty-six participants with note 1 were assigned to 3D printed skull group (3D group), 27 with note 2 to the cadaveric skull group (cadaver group), and 26 with note 3 to the 2D atlas group (atlas group). All participants finished pre-tests to record baseline data. They were administered a 30-min introductory lecture on basicranial anatomy by a third-party non-investigator. During the lecture, cadaveric, 3D printed and 2D atlases of skulls were allocated to the three groups, respectively, with five to six participants using a single model or single set of atlas. Five cadaveric skulls, five 3D printed skulls, and five sets of 2D atlases were used. Each participant received a single printout of teaching materials for note-taking. After the introductory lecture, three groups were assigned to three separate rooms for a 30-min self-directed learning session using cadaveric skulls, 3D printed skulls and 2D atlases, respectively. Exam proctors were assigned to each room to prevent inter-group communication, and they would not answer questions of any participants nor provide any suggestions related to skull anatomy. Learning outcomes were evaluated by post-test immediately after self-directed learning session. All types of learning materials were removed before commencing the post-test. After post-test, each participant finished a subjective evaluation questionnaire. Additionally, participants in the 3D group completed another questionnaire to evaluate the 3D printed skull model.

### Design of learning tasks, test questions and subjective evaluation questionnaire

Learning tasks and test questions were designed according to the teaching syllabus of basicranial anatomy by a non-investigator from a third party. Learning tasks included basic classification of facial bones and cranial bones, cranial sutures, fossae, perforating canals, sulci and grooves on inner and outer face of skulls. Post- and pre-tests composed of a same set of theory test and lab test. The theory test included 18 multiple-choice questions, which mainly covered basic knowledge of skull, including questions such as ‘how many bones skull consists of’, ‘classification of facial bones and cranial bones’, and ‘border of middle cranial fossa and posterior cranial fossa’. Each correct answer to multiple-choice question was awarded one point, and thus the full score of theory test was 18 points. Participants were allowed 15 min to complete theory test. The lab test consisted of 30 labeled structures to be recognized. The 30 structures were equally distributed to cadaveric skulls, 3D printed skulls and 2D atlases. Each learning material comprised 10 labeled structures, mainly evaluating spatial understanding and structure identification. Participants rotated through these 30 structures to write the name of each structure down, spending 45 seconds at each. Each correct answer to structure-recognition was awarded one point, and thus the full score of lab test was 30 points. Full total score of lab and theory test was 48 points. All the test questions are available as Supplementary file 1.

To assess potential efficacy other than objective learning efficiency based on test scores, a questionnaire was designed for subjective evaluation (Table 1). The questions were based on several previous studies to evaluate the role of other 3D printed models^7, 16^. Subjective questionnaires in this trial consisted of five parts, including enjoyment, learning efficiency, authenticity, attitude and intention to use, and used standard five-point Likert-scale to quantify responses (1-strongly disagree, 5-strongly agree with the statement).

**Table 1:** Subjective evaluation questionnaire (1-strongly disagree, 5-strongly agree).

Evaluation to the learning material they used	
Enjoyment	① I enjoyed studying with the help of the learning material in our group very much.
	② It aroused my interests in anatomy study.
Learning Efficiency	① It aided in memorizing.
	② It aided in spatial comprehension.
Authenticity	It present authentic and integral basal cranial features.
Attitude	It’s the best materials in all three groups.
Intention to use	It should be promoted to standard basal cranial anatomy education.
Evaluation to the learning material they used	
Usefulness	3D printed models are highly useful in anatomy education.
Ethics	3D printed models solve the ethical problems brought by cadaver-based anatomy education.
Intention to use	① I’m glad to participate in more educational activities about 3D printed models
	② 3D printed models should be promoted to standard anatomy education.

### Data collection and marking

Demographic information including age and gender was collected during the trial. Participants indicated their group and within-group individual numbers on the pre- and post-test answer sheet. During marking, grouping information was sealed to avoid bias, and each answer sheet was scored and double-checked by two investigators (Pan Z and Wu Y). The counselor provided all the previous academic records of the participants. The files were deleted immediately after data collection to avoid information leakage. Based on pre-test scores of 5 or more, we divided the students into two groups, designated as prepared group (N = 40) and non-prepared group (N = 39). We hypothesized that participants in the former group had prepared anatomical knowledge before the trial, while the latter had not. We selected “5” as the limit since the theoretical average score of 18 multiple-choice questions was 4.5 (four choices per question).

### Statistical analysis

Continuous variables were expressed as median (interquartile range, [IQR]), and categorical variables as number (%). A p-value of <0.05 represented significance. Statistical analysis was performed using IBM SPSS statistical package, version 22 (IBM Corp, Armonk, NY).

Between-group differences in pre- and post-test scores were tested using Kruskal-Walis H test. If there was a significant difference with Kruskal-Walis H test, Mann-Whitney U was employed to pairwise comparison. Comparison between prepared and non-prepared participants, and between male and female participants was performed with Mann-Whitney U test. Categorical variables were compared with chi-square test.

## Results

### Participant demographics

A total of 79 third-year medical students (45 females, 59.21%) attended the study. Following randomization, 27 participants were assigned to cadaveric skull group, 26 to 3D printed skulls group, and 26 to 2D atlas group. All the participants completed the whole trial. There were no statistically significant differences in gender (p = 0.920), age (p = 0.863), or academic ranking in pre-medical study at Tsinghua University Beijing (p = 0.530) (Table 2). None of the participants reported any medical experience outside of the prescribed medical curriculum.

**Table 2:** Between groups values for age, gender, previous academic achievements and pre- and post-test scores.

	Cadaveric skulls (N = 27)	3D printed skulls (N = 26)	Atlas (N = 27)	p-value
**Gender [n(%)]**				
Male	11 (40.74%)	12 (46.15%)	11 (42.31%)	0.920^a^
Female	16 (59.26%)	14 (53.85%)	15 (57.69%)	
Age [Median (IQR)]	21 (20–21)	20 (20–21)	20 (20–21)	0.863^b^
Previous academic achievements [Median (IQR)]	87.38 (84.77–89.22)	86.59 (83.62–88.88)	86.09 (82.62–88.03)	0.530^b^
**Pre-test score [Median (IQR)]**				
Total	5 (4–7)	5 (1.75–7)	3.75 (1.75–6)	0.180^b^
Theory test	5 (3–7)	5 (1–7)	3.5 (1–6)	0.132^b^
Lab test	0 (0–1)	0 (0–1)	0 (0–0.25)	0.895^b^
**Post-test score [Median (IQR)]**				
Total	29.5 (25–33)	31.5 (29–36)	27.75 (24.125–32)	0.044^b,^*
Theory test	15 (13–15)	15 (13.75–16)	14 (13–15.25)	0.699^b^
Lab test	14 (10.5–18)	16.5 (14.375–21.625)	14.5 (10–18.125)	0.049^b,^*
**Change in score [Median (IQR)]**				
Total	25.5 (18–28.5)	27 (23–31.625)	25 (21.75–28.125)	0.163^b^
Theory test	9 (8–12)	9.5 (7.75–13.25)	11.5 (8–13)	0.336^b^
Lab test	13.5 (10.5–18)	16.5 (13.375–21.125)	13.75 (9.75–18.125)	0.046^b,^*

Full scores of theory test, lab test, and total score were 18, 30, and 48 points, respectively.

^a^Chi-square test.

^b^Kruskal-Walis H.

*p < 0.05.

### Comparison of pre- and post-test scores and score changes between three groups

Each score included a sub-score based on theory test and a sub-score involving lab test. Full scores of theory test, lab test, and total score were 18, 30, and 48 points, respectively. Within-subject analysis showed overall improvement in test scores before and after exposure, which were statistically significant for participants in all the three groups (p < 0.001).

#### Pre-test scores

Analysis of pre-test scores revealed no statistically significant differences between the three groups (p = 0.180, 0.132, 0.895 in total score, multiple-choice question, and labeled structure recognition, respectively) (Table 2).

#### Post-test scores

**Figure 4:**
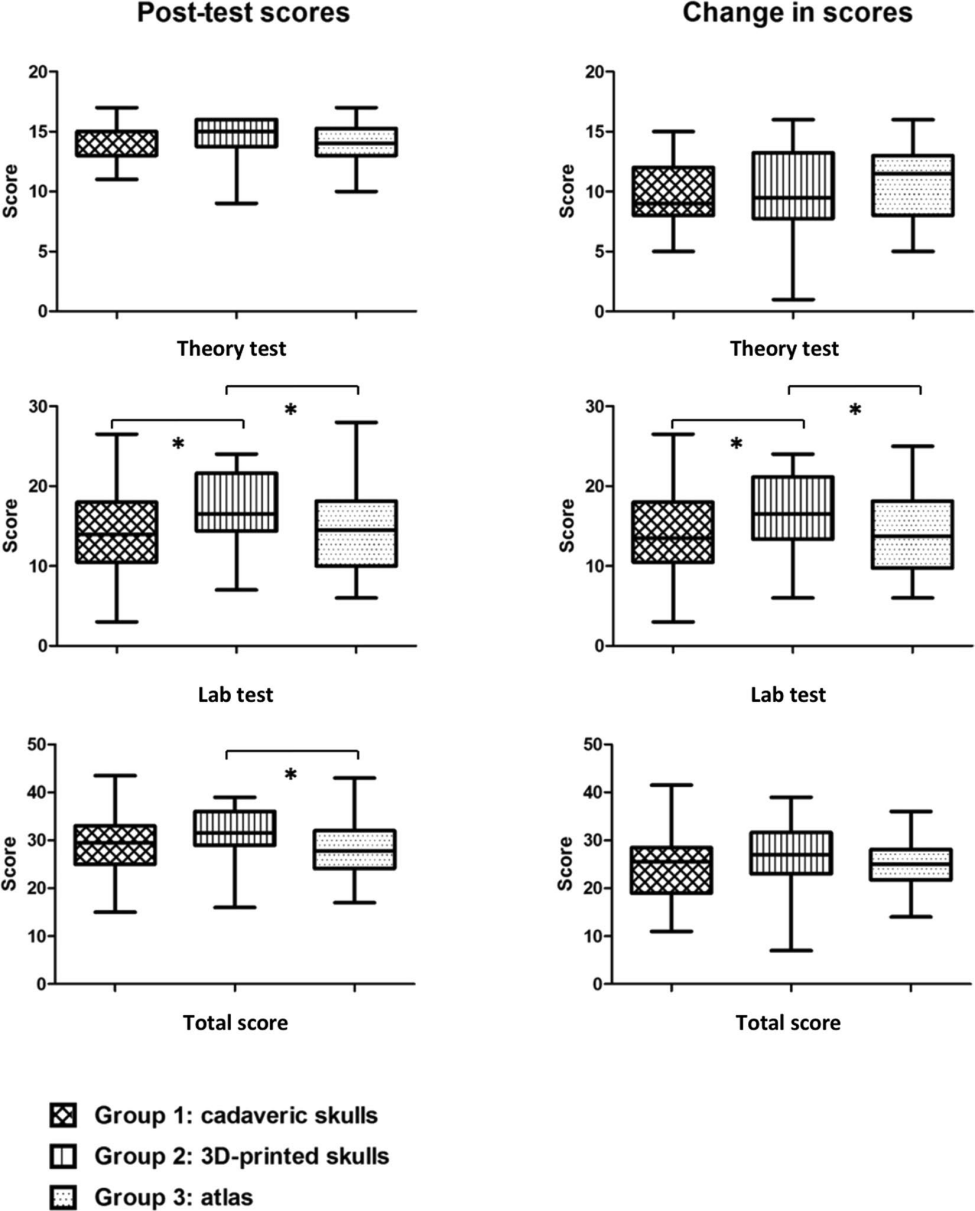
Comparison of three groups in post-test score and change in score. **Theory test** (**full score**: **18**): no significant difference in post-test score or change in score between three groups. **Lab test** (**full score**: **30**) significantly different between three groups in post-test score and change in score (Kruskal-Walis H). Participants in the 3D printed skull group performed better than the other two groups considering post-test score and change in score, while there was no significant difference between cadaveric and atlas group (Mann-Whitney). **Total score** (**full score**: **48**): Significant difference in post-test scores but not in change in scores between three groups (Kruskal-Walis H). 3D printed skull model group performed better than atlas group in post-test scores; however, no significant difference was found in other comparisons (Mann-Whitney). *p < 0.05. Specific p-values are listed in Table 2.

After lectures and small group discussions, Kruskal-Walis H analysis revealed statistically significant differences in total score (cadaver: 29.5 [IQR: 25–33], 3D: 31.5 [IQR: 29–36], atlas: 27.75 [IQR: 24.125–32]; p = 0.044) and structure recognition scores (cadaver: 14 [IQR: 10.5–18], 3D: 16.5 [IQR: 14.375–21.625], atlas: 14.5 [IQR: 10–18.125]; p = 0.049) between the three groups. However, no statistically significant differences were seen in post-test scores of multiple-choice questions (cadaver: 15 [IQR: 13–15], 3D: 15 [IQR: 13.75–16], atlas: 14 [IQR: 13–15.25]; p = 0.699) (Table 2). Pairwise comparison of three groups based on Mann-Whitney U revealed that participants in the 3D printed skull model group performed better than in the other two groups based on the scores involving structure recognition questions (cadaveric vs. 3D, p = 0.034; 3D vs. atlas, p = 0.031). By contrast, no significant difference existed between cadaveric and atlas groups (p = 0.929). The 3D printed skull model performed better than the atlas group based on total scores (p = 0.016). However, no significant difference was seen between cadaveric and atlas groups (p = 0.776), or between 3D and cadaveric groups (p = 0.061) (Fig. 4).

#### Changes in scores

The difference in the post-test and pre-test scores of individual students was designated as change in scores. Kruskal-Walis H analysis revealed that changes in scores involving structure recognition were significantly different between the three groups (cadaver: 13.5 [IQR: 10.5–18], 3D: 16.5 [IQR: 13.375–21.125], atlas: 13.75 [IQR: 9.75–18.125]; p = 0.046). However, changes in total score (cadaver: 25.5 [IQR: 18–28.5], 3D: 27 [IQR: 23–31.625], atlas: 25 [IQR: 21.75–28.125]; p = 0.336) and scores involving multiple-choice questions (cadaver: 9 [IQR: 8–12], 3D: 9.5 [IQR: 7.75–13.25], atlas: 11.5 [IQR: 8–13]; p = 0.163) were not (Table 2). Pairwise comparison of structure recognition scores in the three groups based on Mann-Whitney testing revealed better performance of participants in the 3D printed skull group compared with the other two groups (cadaver vs. 3D, p = 0.033; 3D vs. atlas, p = 0.033), as shown in Fig. 4.

### Comparison of scores between sub-groups

Based on 5 or more pre-test scores involving multiple-choice questions, we divided the students into a prepared group (N = 40) and a non-prepared group (N = 39). We speculated that students with a score of 5 or more had previewed anatomy books or other learning resources before the trial, while the others had not. In addition, we compared the scores between genders.

#### Comparison of scores between prepared and non-prepared participants

**Figure 5:**
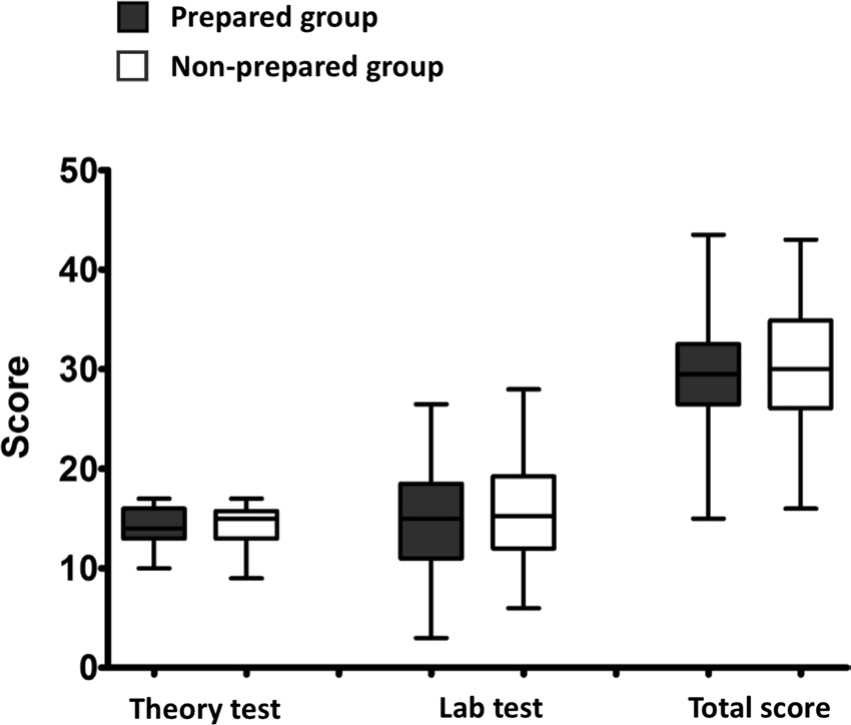
Comparison of post-test scores between prepared and non-prepared participants. No significant difference was found (Mann-Whitney).

Statistically significant differences were found in pre-test total score (p < 0.001) and changes in score (p = 0.008) between the two groups. In post-test, no statistically significant differences were found between the two groups in total score (prepared: 30 [IQR: 26.125–34.875], non-prepared: 29.5 [IQR: 26.5–32.5]; p = 0.655), multiple-choice (prepared: 15 [IQR: 13–15.75], non-prepared: 14 [IQR: 13–16]; p = 0.924) and structure recognition (prepared: 15.25 [IQR: 12–19.25], non-prepared: 15 [IQR: 11–18.5]; p = 0.753), as shown in Fig. 5.

#### Comparison of scores between male and female participants

No differences between males and females were observed in any scores. All gender-based scores were statistically similar in the three groups (Supplementary file 2).

### Response to subjective assessments

**Figure 6:**
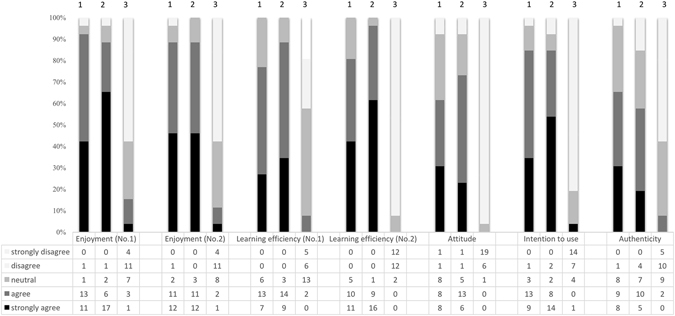
Constitutional ratios of answers to subjective questionnaires. Column 1, 2, and 3 represent cadaveric skulls group, 3D printed skulls group, and atlas group, respectively. The darker the color is, the higher agreement to the statements in questionnaire. Detailed questions of subjective questionnaire shown in Table 1.

Subjective evaluation questionnaire and a comprehensive breakdown of all questions are shown in Table 1. The constitutional ratios of responses between the three groups are compared in Fig. 6. Overall, the responses of 3D and cadaveric skull groups were more positive than in the atlas group. Positive feedbacks (strongly agree, agree, and neutral) exceeded 85% in the 3D and cadaveric skull groups in every question. By contrast, positive feedbacks were less than 45% in the atlas groups except for the response to first question involving learning efficiency (55.7%).

**Figure 7:**
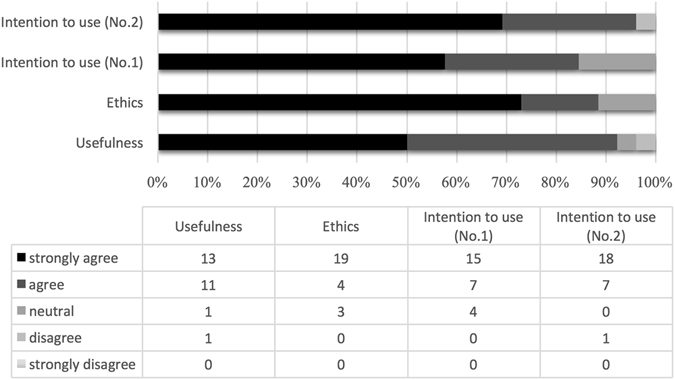
Constitutional ratios of evaluation to 3D-printed skulls. The darker the color is, the higher agreement to the statements in questionnaire. Detailed questions of subjective questionnaire shown in Table 1.

In the subjective evaluation of 3D printed models by the 3D group, all the participants responded “neutral”, “agree” or “strongly agree” to every question except for one “disagree” to the usefulness and one “disagree” to question 2 of “intention” to use (Fig. 7).

## Discussion

Learning anatomy from cadaveric dissection is common in traditional medical education. However, increasing ethical concerns prevent some pre-clinical students from obtaining adequate experience based on cadaveric dissection^1^. 3D printing can serve as an ideal complement to cadaver studies, to avoid challenges involving specimen acquisition, sanitation and ethics. 3D printing is a cost-effective and convenient tool. Nevertheless, most of the available 3D printed products have not been supported by strong evidence for teaching. RCT data comparing 3D prints with cadaveric applications in anatomy education are limited^13–15^.

Major strengths of this study include the stringent experimental conditions. First, interventions were randomly allocated. Statistical analysis showed the absence of inter-group differences in gender, age, and previous academic ranking across 3 groups. Second, we maximized the possible “blindness” in this trial. Lecturers and examination providers were members of a third party organization, blinded to the identity or design of the study, and had no conflict of interest or concern with the study design. Grouping information was concealed to avoid bias during marking. Two investigators reviewed the answers and scores twice. Third, the timing of this trial as well as the educational content coincided with the syllabus. The study was announced by grade counsellor three days before the trial, and nearly the whole class (79 of 80 students) participated in the study voluntarily, with no dropouts. Fourth, both objective and subjective assessments were adopted. In addition, the examination comprised a lab test and a theory test, according to the format of the two major traditional question types in the anatomy curriculum of PUMC. We designated this design as “various question types (VQT)”, and recommend that future studies adopt this model. The subjective questionnaire was designed according to several high-efficiency models reported in previous studies^7, 16^.

All participants attained a basic knowledge of skull anatomy after the study, which suggested that the introductory lectures and group discussions were effective. The post-test total scores showed that the 3D printed model facilitated the learning of skull anatomy compared with traditional atlas and cadaveric skull models. The three groups differ significantly in their ability for structure recognition, while post-test theory test scores were not significantly different, which was possibly because structure recognition questions required the ability to build relations between structures in atlas and reality, the main difficulty in anatomy study, while theory test focused on memorization of theoretical knowledges. With respect to practical learning in anatomy, which is distinct from theoretical knowledge, cadaveric skull and 3D printed skulls were superior in determining spatial relationships and assisting students in quick learning of difficult anatomical structures^17, 18^. In the study of anatomy, structure recognition outweighs theoretical knowledge, which in turn demonstrates the original intent of the study, to build a 3D skull model to assist learning of sophisticated anatomy structures in a relative cheap, convenient and easily accessible way. Recent studies suggest that in the learning of complicated and detailed structures such as middle ear^19^, orbital cavity^20^, multi-component temporal bones^21^, ventricular structures^7^ and teeth^22^, the 3D model played an important role. Medical students, surgeons, and educational experts approved the reliability and utility of models in anatomy and surgical training. Furthermore, three RCTs have been conducted to demonstrate the role of 3D printed models in the study of spinal fractures^13^, cardiac anatomy^14^ and hepatic segment anatomy^15^. Our findings not only provide robust evidence to support the educational efficacy of 3D printed models, but also emphasize their major role as aids to understand and memorize spatial structures practically.

The cadaveric skull group showed no difference from atlas group in total and lab test scores, and the 3D group performed better than cadaver group in total and lab test scores. The results are inconsistent with our hypothesis that 3D printed skull and cadaveric skull groups were approximately equally better than the atlas group, since cadaveric models were found superior to atlas models in several previous studies^13, 23, 24^. This discrepancy was partially attributed to structural variation and damaged structures in the five cadaveric skulls in this trial due to preservation as explained in the Methods section (Fig. 2). It’s noteworthy that the results only showed the 3D printed skulls were better than the partially damaged cadaveric skulls, but not necessarily better than perfectly preserved skulls. Nevertheless, 3D printed skulls help solve the problem that damaged cadaveric skulls may lower the education efficiency, which is a quite common phenomenon due to the difficulties in preservation^3^. The final products of 3D printing resemble 3D atlas and may be somewhat better than the cadavers. The results underscore one of the advantages of 3D printing in that missed or damaged structures are easily restored using commercial software available, and 3D printing can serve as reproducible and standardized 3D references for anatomy education. In addition, several other explanations for the discrepancy in scores are possible. First, cadaveric skulls may trigger negative psychological reaction in participants, facing their initial encounter with cadaver during this trial. The attitudes and psychological stress of medical students in response to dissection are discussed in previous studies^25–27^. Second, motivation plays an important role in study. Novel interventions usually arouse participants’ curiosity and lead to better results^28^. In addition, the atlas group tended to study harder to attain a higher score in post-test as they realized the they missed the opportunity to study with real or 3D printed skulls in this trial^28^.

The results suggest several minor findings. First, the prepared group and the non-prepared group scored almost equal in post-test regardless of the learning material used. The 30-min introductory lecture followed by 30-min group discussion narrowed the differences in preparation. Second, sex-related differences in spatial skills have been reported frequently in studies. Mental rotation, which is closely associated with spatial imagination, shows large sex-related differences^29^. Li *et al*. reported that males have an unfair advantage over females in understanding virtual images. However, this sex-related difference was not observed in the 3D printed model group^13^, suggesting that real models facilitate understanding of spatial structure by females. However, no significant differences existed in spatial imagination between genders in our study. A possible explanation may be that the trial conducted in the top medical college in China, including students with a high-level learning capacity that diminished the difference.

After the trial, subjective evaluation of learning materials in the three groups revealed a positive attitude toward efficacy of 3D printed and cadaveric skulls, but not atlases. Responses indicated that both groups were very satisfied during the trial and unanimously agreed with the learning efficiency of the two formats. Scoring for authenticity was similarly high. Both groups were highly enthusiastic in promotion of 3D skulls in anatomy education. Similarly, in another study comparing different learning materials, medical students selected dissection as the best learning approach to anatomy, while traditional 2D teaching methods such as lectures and textbooks were the least popular^17^. Additionally, the results not only indicated approval of 3D printing in anatomy, but also highlighted the role of 3D printing in addressing ethical issues associated with cadavers. Notably, participants in the cadaveric group reported damaged structures and variations in cadaveric skulls, which was a limitation of cadaveric skulls.

The characteristic study design of this trial is defined as follows: “immediate education effect examination (IEEE)” with “various question types (VQT)”, in which the study efficiency was reviewed immediately after various interventions, and both multiple-choice and labeled recognition question were used in evaluation. We recommend that RCT studies in the future investigate the efficiency of 3D printed models using a VQT design, focusing on different learning abilities. Additional RCTs, both in IEEE pattern or “long-term education effect examination (LEEE)” pattern, are needed to confirm the efficiency of high-quality 3D printed models and their possible limitations. Future studies investigating the role of 3D printing in medical education should include participants from different grades and medical colleges, and employ emerging virtual reality as additional educational interventions.

The study limitations are as follows. First, we recruited participants at a certain grade in the PUMC who finished their pre-medical course at Tsinghua University (Beijing). The students were highly skilled and competent in Mathematics^30^ and Physics. The sample itself may be a source of bias, especially when spatial imagination and learning efficiency are considered. Above-average learning ability may diminish the differences between groups, possibly suggesting the absence of significant differences between cadaver and atlas groups, and the absence of differences when comparing multiple-choice questions and sex-related differences. In addition, we were unable to enlarge our sample size to include medical students at different levels subject to experimental conditions. Further studies investigating medical students from diverse educational backgrounds are needed. Second, no preliminary experiments were conducted prior to our study, due to the limited number of students available. We ensured that enough participants were enrolled in the formal experiment by avoiding any preliminary experiments. Thirdly, because of the unique experimental features in medical education, our participants required access to interventions represented by different learning aids in our study. Although we failed to disclose the purpose of our study in advance, or officially informed them of the experimental materials, the study design does not represent a single- or double-blinded trial. Knowledge of the grouping and interventions affected students’ performance partially. We obtained feedback from a few participants later, which suggested that most of them could not distinguish a 3D printed skull from a cadaveric skull. Finally, five cadaveric skulls are different in size and are not intact, while the five 3D printed skulls are uniform. This RCT cannot demonstrate the education difference between perfectly-preserved cadaveric skulls and 3D printed skulls. In addition, the 3D printed skulls did not mimic the “dissection” feature of cadavers, which could be improved in the next version.

## Conclusion

3D printed skulls facilitate basicranial education, especially in assisting structure recognition, compared with cadaveric skulls and atlas. Other advantages over cadavers relate to ethics, cost, hygiene and repaired fragile structures. Additional RCTs, both in IEEE or LEEE format, preferably using a VQT model, are needed to validate 3D printing in medical education.

## Electronic supplementary material

Supplementary information
