# Supplementary material for: The role of three-dimensional printed models of skull in anatomy education: a randomized controlled trail
Source: Sci Rep. 2017 Apr 3;7:575. doi: 10.1038/s41598-017-00647-1 (PMC5428829; doi:10.1038/s41598-017-00647-1)
Supplement: Supplementary file 1 — Supplementary information [file 41598_2017_647_MOESM1_ESM.pdf]

The role of three-dimensional printed models of skull in anatomy education: a randomized controlled trail

Shi CHEN[1,2+], Zhouxian PAN[3,+], Yanyan WU[3], Zhaoqi GU[3], Man LI[3], Ze LIANG[3], Huijuan ZHU[1], Yong YAO[4], Wuyang SHUI[5], Zhen SHEN[6], Jun ZHAO[7], Hui PAN [1,2,7*]

[1]Department of Endocrinology, Endocrine Key Laboratory of Ministry of Health, Peking Union Medical College Hospital (PUMCH), Chinese Academy of Medical Sciences & Peking Union Medical College (CAMS & PUMC), Beijing 100730, China

[2]National Virtual Simulation Laboratory Education Center of Medical Sciences, PUMCH, CAMS & PUMC, Beijing 100730, China

[3]Eight-year Program of Clinical Medicine, PUMCH, CAMS & PUMC, Beijing 100730, China

[4]Dept. of Neurosurgery, PUMCH, CAMS & PUMC, Beijing 100730, China

[5]College of Information Science and Technology, Beijing Normal University, Beijing 100875, China

[6]Institute of Automation, Chinese Academy of Sciences (CASIA), Beijing 100190, China

[7]Dept. of Education, PUMCH, CAMS & PUMC, Beijing 100730, China

*corresponding author: panhui20111111@163.com

[+]these authors contributed equally to this work

Supplementary file 1

QUESTIONS: Test on basicranial anatomy

1. Multiple choice

   Directions: For each question, you must choose the best answer from the four choices marked A, B, C and D.

No.	Questions	Answers
1)	How many pieces of cranial bones do human have (including bones of facial cranium and cerebral cranium)?<br>A.18 B.20 C.23 D.28	
2)	Which of the following **does not** belong to bones of cerebral cranium?<br>A.frontal bone B.zygomatic bone C.parietal bone D.ethmoid bone	
3)	Which of the following does not appear in pair?<br>A.parietal bone B.occipital bone C.temporal bone D.zygomatic bone	
4)	Lambdoid suture is composed of ____ and ____.<br>A.parietal bone, occipital bone B.parietal bone, frontal bone<br>C.frontal bone, sphenoid bone D.left parietal bone, right parietal bone	
5)	Infraorbital foramen is located on____.<br>A.maxilla B.zygomatic bone C.nasal bone D.mandible	
6)	Hypophysial fossa is located on____.<br>A.lamina cribrosa B.orbital plane of frontal bone<br>C.petrosal bone D.body of sphenoid	
7)	Which of the following structure does not form anterior cranial fossa?<br>A.orbital plane of frontal bone B.lamina cribrosa<br>C.great wing of sphenoid bone D.lesser wing of sphenoid bone	
8)	Pterion is composed of____<br>A.frontal bone, sphenoid bone, parietal bone and temporal bone<br>B.frontal bone, occipital bone, parietal bone and temporal bone<br>C.zygomatic bone, sphenoid bone, parietal bone and temporal bone<br>D.zygomatic bone, frontal bone, occipital bone and temporal bone	
9)	Which sentence about mandible is **false**?<br>A.includes rami mandibulae and corpus mandibulae<br>B.angulus mandibulae is palpable from body surface<br>C.foramina mandibulae are lateral to rami mandibulae<br>D.foramina mentale are anterior lateral to corpus mandibula	
10)	Which of the following structure is located in middle cranial fossa?<br>A.internal acoustic pore B.trigeminal impression<br>C.clivus D.petro-occipital fissure	
11)	Which of the following structure is connected with carotid canal?<br>A.foramen lacerum B.foramen ovale<br>C.petro-occipital fissure D.foramen spinosum	
12)	Which of the following structure **is not** located on great wing of sphenoid bone?	

	A.foramen spinosum B.foramen ovale C.foramen rotundum D.optic canal	
13)	The foramen connecting basis cranii with nasal cavity is_____<br>A.optic canal B.foramina ethmoidale<br>C.foramen rotundum D.foramen spinosum	
14)	Which of the following structure **is not** located in posterior cranial fossa?<br>A.internal acoustic meatus B.foramen lacerum<br>C.hypophysial fossa D.jugular foramen	
15)	Which of the following structure **is not** located on the body of sphenoid bone?<br>A.optic canal B.superior orbital fissure<br>C.tuberculum sellae D.chiasmatic sulcus	
16)	Which of the following structure **is not** located on occipital bone?<br>A.hypoglossal canal B.foramen magnum<br>C.groove for sigmoid sinus D.sinus jugularis	
17)	Which of the following structure **is not** located on occipital bone?<br>A.mastoid foramen B.occipital condyle<br>C.hypoglossal canal D.clivus	
18)	Which piece of bone is connected with all cranial bones?<br>A.ethmoid bone B.sphenoid bone C.zygomatic bone D.parietal bone	

2. Lab test

Directions: Questions A1-10 are all structures on 3D printed skulls. B1-10 are structures on cadaveric skulls. C1-10 are structures on atlas.

A1		B1		C1	
A2		B2		C2	
A3		B3		C3	
A4		B4		C4	
A5		B5		C5	
A6		B6		C6	
A7		B7		C7	
A8		B8		C8	
A9		B9		C9	
A10		B10		C10	

ANSWERS: Test on basicranial anatomy

1. Multiple choice

No.	1)	2)	3)	4)	5)	6)	7)	8)	9)
Answers	C	B	B	A	A	D	C	A	C
No.	10)	11)	12)	13)	14)	15)	16)	17)	18)
Answers	B	A	D	B	B/C	B	C	A	B

2. Lab test

A1	Superior orbital fissure	B1	Foramen lacerum	C1	Lesser wing of sphenoid bone
A2	Foramen ovale	B2	Foramen spinosum	C2	Internal occipital Protuberance
A3	Foramen magnum	B3	Hypoglossal canal	C3	Groove for sigmoid sinus
A4	Jugular foramen	B4	Internal acoustic meatus	C4	Jugular groove
A5	Hypophysial fossa	B5	Anterior clinoid process	C5	Foramina ethmoidale or cribriform plate
A6	Optic canal	B6	Crista galli	C6	Occipital condyle
A7	Foramen lacerum	B7	Foramen spinosum	C7	External occipital protuberance
A8	Mastoid process	B8	Carotid canal	C8	Foramen magnum
A9	Jugular foramen	B9	Foramen ovale	C9	Pharyngeal tubercle
A10	Inferior orbital fissure	B10	External acoustic canal	C10	Medial plate of pterygoid process

Supplementary file 2

Comparison between males and females

		Male N=34	Female N=45	p
**Total score [Median (IQR)]**	Post-test	29.5(25.875-34.5)	29.5(26.75-33.25)	0.659
	Difference between pre- and post-test	23.5(19.375-29.125)	26(23-28.5)	0.184
**Score of multiple choice question [Median (IQR)]**	Post-test	15(13-15)	14(13-16)	0.716
	Difference between pre- and post-test	8(7-12)	11(8-13)	0.029
**Score of structure recognition [Median (IQR)]**	Post-test	15.25(10.5-18.625)	15(12-18.75)	0.747
	Difference between pre- and post-test	14.25(10.5-18.5)	15(11.75-18.25)	0.641
